# PDDD-PreTrain: A Series of Commonly Used Pre-Trained Models Support Image-Based Plant Disease Diagnosis

**DOI:** 10.34133/plantphenomics.0054

**Published:** 2023-05-18

**Authors:** Xinyu Dong, Qi Wang, Qianding Huang, Qinglong Ge, Kejun Zhao, Xingcai Wu, Xue Wu, Liang Lei, Gefei Hao

**Affiliations:** ^1^ State Key Laboratory of Public Big Data, College of Computer Science and Technology, Guizhou University, Guiyang 550025, China.; ^2^ The School of Physics and Optoelectronic Engineering, Guangdong University of Technology, Guangzhou 510006, China.; ^3^ National Key Laboratory of Green Pesticide, Key Laboratory of Green Pesticide and Agricultural Bioengineering, Ministry of Education, Guizhou University, Guiyang 550025, China.

## Abstract

Plant diseases threaten global food security by reducing crop yield; thus, diagnosing plant diseases is critical to agricultural production. Artificial intelligence technologies gradually replace traditional plant disease diagnosis methods due to their time-consuming, costly, inefficient, and subjective disadvantages. As a mainstream AI method, deep learning has substantially improved plant disease detection and diagnosis for precision agriculture. In the meantime, most of the existing plant disease diagnosis methods usually adopt a pre-trained deep learning model to support diagnosing diseased leaves. However, the commonly used pre-trained models are from the computer vision dataset, not the botany dataset, which barely provides the pre-trained models sufficient domain knowledge about plant disease. Furthermore, this pre-trained way makes the final diagnosis model more difficult to distinguish between different plant diseases and lowers the diagnostic precision. To address this issue, we propose a series of commonly used pre-trained models based on plant disease images to promote the performance of disease diagnosis. In addition, we have experimented with the plant disease pre-trained model on plant disease diagnosis tasks such as plant disease identification, plant disease detection, plant disease segmentation, and other subtasks. The extended experiments prove that the plant disease pre-trained model can achieve higher accuracy than the existing pre-trained model with less training time, thereby supporting the better diagnosis of plant diseases. In addition, our pre-trained models will be open-sourced at https://pd.samlab.cn/ and Zenodo platform https://doi.org/10.5281/zenodo.7856293.

## Introduction

Plant disease diagnosis has played an important role during crop production. In 2050, according to UN FAO statistics [1], the world’s total crop production will reach 9.4 billion tons, and the world’s population is expected to reach 9.1 billion people. To address the nutritional needs of such a large number of people, the growth rate of food should increase to 70% by 2050 [2]. Plant diseases are one of several factors that act as a constraint on increasing food production and seriously jeopardize the world’s ability to feed itself by lowering crop yields. According to statistics, plant diseases account for 20% to 40% of the global decline in food production [3]. Therefore, prompt detection of plant diseases is essential for managing and making decisions regarding agricultural production because it can prevent the disease’s full spread, enable early treatment, and increase crop yields [4]. Generally speaking, a diseased plant leaves visible traces or lesions on its leaves, flowers, and fruits. Additionally, each disease leaves the plant with distinct and noticeable symptoms that can be used to identify the type of plant disease. Since most plant disease symptoms are found on the leaves [5], the leaves are the primary source of identification for existing plant diseases. More and more farmers adopt this way to detect plant disease to boost food production.

Automated methods of plant disease detection are becoming a hot topic of research, saving enormous costs. There are 2 traditional ways to identify plant leaves. One is through the training of experts in the identification of leaves. The other is the use of machine learning methods for image processing to identify the leaves [6]. The former requires trained experts to identify diseased leaves by visually examining the surface morphology of plant leaves and analyzing the presence of diseased leaves one by one. This manual operation is time-consuming, laborious, costly, and inefficient, and there is also a risk of errors due to subjective perception [7]. With the rise of machine learning methods, especially the introduction of deep learning methods [8–12], computer vision has made an enormous leap. This method is also adopted in recognizing and detecting plant diseases because the deep learning-based model can effectively extract high-level semantic and low-level detailed information [13] of images as meaningful disease feature representations [14,15].

The convolutional neural network (CNN), one of the main deep learning methods, has gradually become the standard method in CNN-based plant disease detection models. Compared to the manual method of operation, the deep learning approach can substantially lower operation costs, improve the effectiveness of recognition and detection, and reduce subjective error. Using a convolution neural network, Sladojevic et al. [16] trained a model that can identify 13 different types of plant diseases, which is also the first to propose a plant disease identification method based on deep learning. Mohanty et al. [17] reported a public dataset of 54,305 diseased and healthy plant leaf images collected under controlled conditions to train a CNN to identify 26 diseases in 14 plants. Brahimi et al. [18] proposed a CNN to train 9 diseases in tomato leaf disease on 14,828 image datasets and achieved excellent results. Lu et al. [19] introduced a CNN to identify 10 common diseases in rice and achieved much higher accuracy than traditional machine learning. However, there are drawbacks to designing CNN structures to diagnose plant diseases, including complex dispositions, poor performance and efficiency, and challenging applications. Therefore, pre-trained models showing excellent computer vision potential are gradually being used to diagnose plant diseases.

**Fig. 1.**
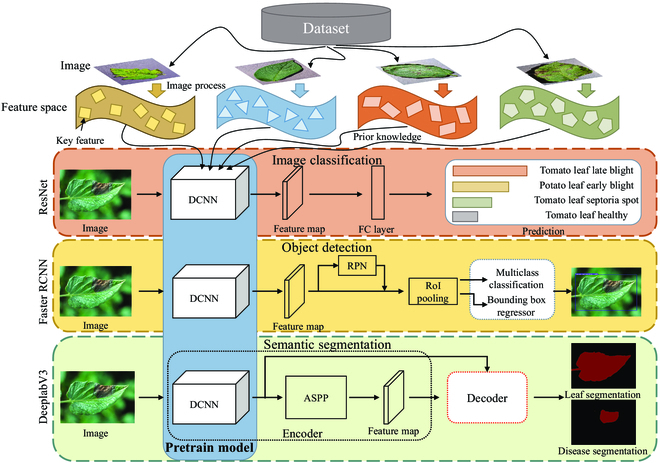
The role of pre-trained models in plant disease diagnosis.

A pre-trained model is the intrinsic core factor that enables the deep learning method to succeed in diagnosing plant diseases, which includes the basic prior knowledge of image processing. As shown in Fig. 1, a pre-trained model is adopted to improve the performance of the target task by pre-training an initial model on the original task and then using that model on the target task to fine-tune the initial model for the characteristics of the target task. The commonly used pre-trained models are from the prior knowledge of ImageNet dataset [20], which has 1,000 classes and uses 1,281,167 images as the training set and 50,000 images as the validation set. As one of the remarkable milestones in deep learning, this dataset has become a benchmark for many computer vision tasks. Pre-trained models based on the ImageNet dataset, like AlexNet [8], VGGNet [9], and ResNet [21], are successful in computer vision and are progressively used to aid in diagnosing plant diseases [22–25] because they can shorten training times and increase training accuracy. For instance, Wang et al. [23] utilized the VGG-16 [9] pre-trained model to diagnose apple black rot disease severity in PlantVillage, which trained an integrated plant disease identification system on a dataset of 87,848 plant diseases containing 25 different plant species. Ferentinos [26] chose various pre-trained models, including AlexNet [8], GoogleNet [10], and VGGNet [9], and achieved a satisfactory performance. Zheng et al. [27] extracted feature maps for strawberry canopy and biomass prediction through a VGG-16 [9] backbone network with a ResNet-50 [21] pre-trained model. Johnson et al. [28] used the ResNet-50 [21] pre-trained model as a backbone network to identify potato wilt spots. Zhang et al. [29] achieved excellent results using Darknet53 [30] as a backbone network for citrus fruit detection and yield estimation. Therefore, with the help of pre-trained models, the precision of plant disease diagnosis methods has reached a new level.

**Fig. 2.**
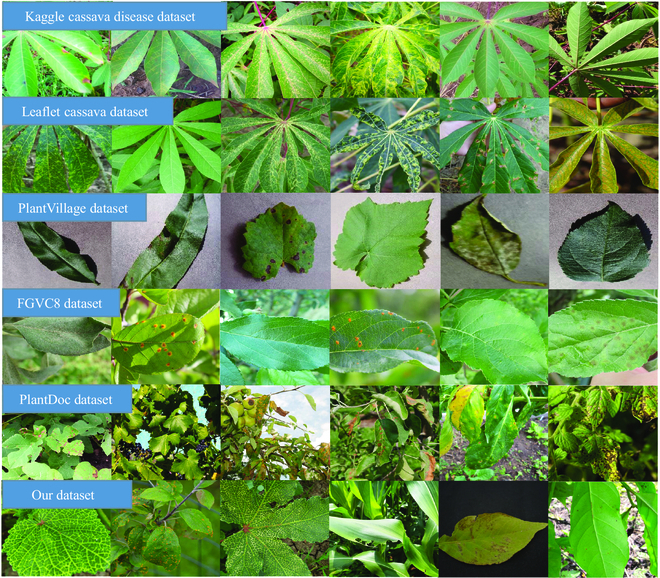
Examples of the different datasets.

However, a pre-trained model based on a large plant disease dataset does not emerge, which cannot bring adequate domain knowledge to improve plant disease diagnosis further. Although deep learning pre-trained model techniques have achieved impressive results in diagnosing plant diseases, it is inappropriate to use the ImageNet or a non-botanical dataset as the pre-trained models for plant disease diagnosis, leading to weak generalization and precision. The cause of this problem is that current pre-trained models do not have domain knowledge of plant disease. Specifically, since most ImageNet or non-botanical classes are unrelated to plant diseases, pre-trained models lack sufficient knowledge of plant phenotypes, which leads to poor accuracy in diagnosing plant diseases in some subclass tasks, like identification, detection, segmentation, and others. Overall, existing studies have yet to discuss this issue in depth from botany.

To this end, we construct a series of commonly used pre-trained models based on a large plant disease dataset to support image-based plant disease diagnosis, named PDDD (plant disease diagnosis dataset). The primary goal of this paper is to propose several faster and more accurate pre-trained models for diagnosing plant diseases. These models are used to improve the performance of current deep-learning techniques for plant disease diagnosis models. First, we collect and compile over 400,000 relevant images of 40 plant species with 120 plant disease classes to form a super-large-scale plant disease dataset. Meanwhile, we adopt existing standard models to pre-train on this dataset to ensure that the pre-trained models gain sufficient expertise about plant diseases. Furthermore, we combine plant diseases with pre-trained models using plant diagnosis for downstream tasks. Additionally, we propose a series of pre-trained models for plant diseases with various structures and parametric quantities to accommodate the various requirements of plant disease diagnosis scenarios and devices. In particular, to demonstrate the excellent performance of the plant disease pre-trained models, we conduct sufficient experiments in plant diseases, such as plant disease identification, plant disease detection, and plant disease segmentation. Finally, extensive experiments show that our plant disease pre-trained models can achieve higher accuracy for plant disease diagnosis in less time. More importantly, for the development of image-based plant disease diagnosis, we make our plant disease pre-trained model an open resource so that all subsequent work can benefit from the plant disease pre-trained model’s convenience.

In summary, the main contributions are concluded as follows:

• We collect a large-scale plant disease dataset of over 400,000 images from 40 plant species in the field and online. In the meantime, we classify these plants into 120 disease categories to train our plant disease pre-trained model.

• We propose a framework for plant disease pre-trained models that enable existing deep learning-based plant disease diagnosis models to gain more relevant knowledge about plant diseases and thus perform better plant disease diagnosis.

• We experiment extensively with the plant diseases’ diagnoses, such as plant disease identification, plant disease detection, and plant disease segmentation, to evaluate the effectiveness of our plant disease pre-trained model.

• We open-source our proposed series of plant disease pre-trained models in the hope that they can better assist in the follow-up of plant disease diagnosis and the development of the plant disease. At the same time, we provide detailed instructions and parameter-setting procedures for the plant disease pre-trained models to help researchers quickly solve their own plant disease diagnosis problems with the help of our pre-trained models.

## Materials and Methods

### Plant disease dataset

A large-scale PDDD is extremely critical for training the pre-train model, which can include sufficient plant disease knowledge and phenotype information. Building high-quality, large-scale plant disease datasets is challenging due to the complexity, diversity, and variability of plant disease occurrence [31]. ImageNet [20], the existing primary pre-trained dataset, contains over one million images for 1,000 classes. A plant disease dataset of roughly the same size as ImageNet is required to train the pre-trained model. However, the current standard plant disease dataset is not large enough, as shown in Table 1 and Fig. 2. The leaflet cassava disease dataset [22] has 1,896 images of 6 cassava diseases, whereas the Kaggle cassava disease dataset [32] has 9,436 images of 5 cassava diseases. PlantVillage [33], the most prominent open-source plant disease identification dataset, contains only 38 plant diseases and 54,309 images. IP102 [34] contains numerous images of plant pests, not plant diseases. Furthermore, due to the limited availability of only a demonstration subset of 50 images per class from PDD271 [31], amounting to 13,550 images in total, we are unable to obtain more reliable information on this dataset. In addition, too much work on plant disease identification and diagnosis has yet to be open-sourced, resulting in a tiny number of existing large-scale plant disease datasets. Therefore, we decide to collect and take photos of plant diseases to form a large-scale dataset to train a plant disease pre-trained model.

**Table 1.** Statistics on existing plant disease datasets.

Dataset	Image number	Class number	Coverage	Note
Leaflet cassava dataset [22]	1,896	6	Only cassava	Not open-source
Kaggle cassava disease [32]	9,436	5	Only cassava	
PlantVillage dataset [33]	54,309	38	Fruit, crop	
Leaf disease dataset [16]	4,483	15	Only fruits	
Apple leaf disease dataset [48]	404	3	Only apple	
Plant pathology 2021 - FGVC8 [36,49]	18,632	12	Only apple	
Crop pests dataset [50]	4,500	40	Crop pest	
IP102 [34]	75,222	102	Crop pest	
PDD271 [31]	220,592	271	Fruit, vegetable, crop	Not Open-Source
PDDD	421,133	120	Fruit, vegetable, crop	

To ensure the acquisition of high-quality images of plant diseases, we adopt a 4-step methodology.

#### Selecting the shooting location

To construct a large-scale, diverse, and broad-coverage plant disease identification dataset, we selected Guiyang City, Guizhou Province, China (106.63E, 26.65N) as the main shooting location because of its subtropical monsoon climate with warm and humid conditions, 4 distinct seasons, abundant sunshine, and sufficient precipitation. These factors provide favorable natural conditions for the growth and development of various plants and their related diseases. We collected images of major food crops such as rice, wheat, and maize, and major fruit and vegetable crops such as apples, grapes, and strawberries at different seasons, weather conditions, angles, and distances. These image data contain both normal leaves and leaves suffering from many common diseases. For some crops that are not or less commonly grown in Guiyang (e.g., citrus, tea, bananas), we collaborated with plant disease teams in the major growing sites of these crops in southern Chinese provinces (e.g., Guangxi, Yunnan, and Hainan) and asked them to take images according to our requirements. In addition to our own image data collection, we also obtained image data from the Internet from a number of open-source datasets that matched or complemented our data in terms of plant species and disease categories. We filtered and processed these data as necessary to increase the richness of our dataset.

#### Establishing a classification system

Considering that different plants exhibit different plant disease patterns and require diagnosis by experts in different fields, we establish a hierarchical classification system based on plant species and common disease types. The system consists of a root catalog of datasets, a catalog of plant species, and a sub-catalog of diseases. For instance, for rice leaf blight, the corresponding directory structure is: /Rice/brown spot/. To ensure that each image in the dataset is accurately and consistently labeled, we invite 7 agricultural experts to participate in the dataset construction process and follow their guidelines for classification and naming. For some disease instances that were unfamiliar to experts or were difficult to distinguish, we simply label them as “diseased” or “healthy” without assigning specific subcategories.

#### Implementing acquisition and photography

The uncertainty and fragmentation of the timing and location of each disease pose a challenge to obtaining timely and adequate image data. Therefore, we employ a combination of online collection and field photography to increase the amount and diversity of data.

Field photography: We divide the participants into 8 groups of 5 people each, and equip them with cameras, positioning locators, laptops, and other equipment. Six of the groups are responsible for field filming at several farms near Guiyang City, with an agricultural expert accompanying them for guidance and on-site quality control. Before filming, we make detailed plans and routes based on the crop lists and distribution maps provided by the farms and investigate the possible dates of different types or degrees of disease occurrence in different crops during the current year. We try to select 3 days within the incidence period for continuous filming. During the shooting process, we attempt to capture images under different angles, distances, and lighting conditions, and record metadata such as time, location, species, and environment corresponding to each image. In this way, we can ensure that the dataset has sufficient quantity, diversity, and accuracy that facilitate data processing and analysis at a later stage.

Online collection: In order to expand the variety and quantity of images and to address the insufficiency of disease categories, we selectively sourced and classified images of various plant species and disease types that were compatible or complementary to our own data from multiple open-source datasets. Our initial focus was on 2 datasets, PlantVillage [33] and fine-grained visual classification (FGVC), which encompass similar or diverse plant and disease categories.

Pennsylvania State University’s PlantVillage dataset is composed of 54,303 images of healthy and diseased leaves, categorized into 38 distinct classes representing 14 crops and 26 diseases. We chiefly utilized their open-source RGB images as they better captured the essential features of plant diseases. In contrast, the University of California, Berkeley’s FGVC dataset is a compendium of several sub-datasets on FGVC. Our attention was drawn to the FGVC7 [35] and FGVC8 [36] datasets, which pertain to apple leaf health assessment and fine-grained recognition. The FGVC7 dataset comprises apple leaf images categorized into 4 classes with a total of 1,821 training images, as well as 1,821 test images, and we only used the annotated training set images. The FGVC8 dataset, a CVPR workshop dataset from 2021, is a collection of 23,000 high-quality RGB images that aims to identify 27 diseases across 10 plant species (apple, cherry, grape, citrus, peach, strawberry, tomato, pepper, corn, and potato) in 61 distinct categories. We selected only the 18,633 disease images that were relevant to apple leaf diseases in this dataset. These 2 datasets provide a fine-grained identification framework for apples and comprise complex plant disease images that feature one or more apple diseases, posing a notable challenge for plant disease diagnosis. In order to enhance the robustness and generalization capability of our PDDD, we incorporated these images into our dataset.

In addition, we have incorporated rare plant disease images from the dataset curated by Chouhan et al. [37]. This dataset consists of 4,503 images, which were collected from Shri Mata Vaishno Devi University, Katra, India during the period of March to May 2019. By merging a total of 79,260 images from the aforementioned 4 publicly available datasets, we have expanded the scope and size of our dataset. Through this endeavor, we strive to improve the generalization ability and diagnostic precision of our pre-trained model across diverse situations. After sorting out according to our statistics, the ratio between field-taken plant disease images versus online-collected ones is about 8:2.

#### Performing calibration and enhancement

After completing the data collection, we verify with 7 agricultural experts that each image is correctly labeled and perform a final cleaning of the dataset to remove images that are blurred, out of focus, or have other distracting factors to improve the quality of the dataset. Moreover, to mitigate the category imbalance problem and to enable the pre-trained model to acquire proper prior knowledge from the dataset, we perform some simple data augmentation operations such as rotation, scaling, cropping, and Gaussian blurring for the categories with fewer images and ensure that each category has at least 100 images. Table 2 represents the number of images for each type of disease in the dataset without data augmentation.

**Table 2.** Table of the number of images for each type of disease in the PDDD dataset.

Plant	Plant diseased name	Image number	Plant	Plant diseased name	Image number	Plant	Plant diseased name	Image number
*Alstonia scholaris*	Diseased	508	Corn	Rust	11,454	Rice	Brown spot	776
	Healthy	358		Gray leaf spot	7,057		Healthy	1,966
Apple	Black rot	7,023		Healthy	12,848		Hispa	565
	Cedar apple rust	5,402		Northern leaf blight	9,200		Leaf blast	1,220
	Complex disease^a^	1,693	Cotton	Diseased	1,259		Neck blast	1,000
	Frog eye leaf spot	3,346		Healthy	1,016	Soya bean	Healthy	14,390
	Healthy	15,841	Cucumber	Diseased	350	Squash	Powdery mildew	6,624
	Powdery mildew	1,271		Healthy	341	Strawberry	Calcium deficiency	805
	Rust	2,802	Guava	Diseased	284		Healthy	6,892
	Scab	13,610		Healthy	554		Leaf scorch	8,117
Arjun	Diseased	464	Grape	Black measles	9,411	Sugarcane	Diseased	491
	Healthy	440		Black rot	8,780		Healthy	488
Bael	Diseased	118		Healthy	5,783	Sunflower	Downy mildew	120
Banana	Cordana	33		Leaf blight	7,879		Healthy	134
	Healthy	128	Hops	Downy	166		Gray mold	72
	Pestalotiopsis	173		Healthy	528		Leaf scars	140
	Sigatoka	473		Powdery	106	Tea	Algal leaf	113
Basil	Healthy	148	Jamun	Diseased	690		Anthracnose	100
Bean	Angular leaf spot	432		Healthy	558		Bird eye spot	100
	Healthy	427	Jatropha	Diseased	248		Brown blight	113
	Rust	436		Healthy	266		Gray light	100
Blueberry	Healthy	5,894	Lemon	Diseased	154		Healthy	74
Cauliflower	Bacterial spot rot	173		Healthy	318		Red leaf spot	143
	Black rot	40	Mango	Diseased	530		White spot	142
	Downy mildew	146		Healthy	340	Tomato	Bacterial spot	11,529
	Healthy	188	Orange	Huanglongbing	15,277		Early blight	8,318
Cherry	Healthy	7,513	Okra	Diseased	1,072		Healthy	10,250
	Powdery mildew	7,708		Healthy	877		Late blight	11,204
Chinar	Diseased	240	Peach	Bacterial spot	12,240		Leaf mold	8,045
	Healthy	206		Healthy	5,767		Mosaic virus	5,832
Citrus	Black spot	318	Pepper	Bacterial spot	8,229		Septoria leaf spot	10,525
	Canker	372		Healthy	9,997		Spider mites	8,198
	Greening	5,794	Pomegranate	Diseased	544		Arget spot	9,238
	Healthy	89		Healthy	574		Yellow leaf curl virus	22,882
Coffee	Healthy	435	*Pongamia pinnata*	Diseased	552	Wheat	Brown rust	902
	Miner	308		Healthy	644		Complex diseased*	12,500
	Red spider mite	167	Potato	Early blight	9,418		Healthy	3,718
	Rust	683		Healthy	5,213		Septoria	97
Corn	Blight	1,336		Late blight	9,376		Stripe rust	208
	Complex diseased*	2,225	Raspberry	Healthy	3,217		Yellow rust	924

a Complex disease means the presence of multiple diseases on the collected leaves at the same time.

**Table 3.** The list of our trained plant disease pre-trained model networks.

Model structure	Parameters	FLOPs	Model structure	Parameters	FLOPs
AlexNet [8]	60M	0.72B	EfficientNetV2-S [51]	22M	8.8B
GoogleNet [10]	5.6M	1.5B	EfficientNetV2-M [51]	54M	24B
VGG-16 [9]	134M	15.3B	EfficientNetV2-L [51]	120M	53B
VGG-19 [9]	144M	19.6B	DarkNet-19 [30]	20.8M	5.6B
ShuffleNet-V1(0.5×) [52]	–	38M	MobileNet-V1 [53]	4.2M	–
ShuffleNet-V1(1x) [52]	–	140M	MobileNet-V2 [54]	3.4M	–
ShuffleNet-V1(2×) [52]	–	524M	MobileNetV3-Small [55]	2.5M	–
ShuffleNet-V2(1×) [56]	2.3M	146M	MobileNetV3-Large [55]	5.4M	–
ShuffleNet-V2(1.5×) [56]	3.4M	299M	ViT-B/16 [57]	87M	56B
ShuffleNet-V2(2×) [56]	7.4M	591M	ViT-B/32 [57]	88M	13B
ResNet-18 [21]	11.2M	1.8B	DeiT-B [58]	86M	18B
ResNet-34 [21]	21.5M	3.6B	ConvNext-T [59]	29M	4.5G
ResNet-50 [21]	26M	3.8B	ConvNext-S [59]	50M	8.7G
ResNet-101 [21]	44.6M	7.6B	ConvNext-B [59]	89M	15.4G
ResNet-152 [21]	60M	11.3B	ConvNext-L [59]	198M	34.4G
DenseNet-169 [42]	14M	3.5B	Swin-T [60]	28M	4.5G
DenseNet-264 [42]	34M	6.0B	Swin-S [60]	50M	8.7G
EfficientNet-B0 [43]	5.3M	0.39B	Swin-B [60]	88M	15.4G
EfficientNet-B1 [43]	7.8M	0.70B	Swin-L [60]	197M	34.5G
EfficientNet-B2 [43]	9.2M	1.0B	RegNetX-200MF [61]	2.7M	0.2B
EfficientNet-B3 [43]	12M	1.8B	RegNetX-400MF [61]	5.2M	0.4B
EfficientNet-B4 [43]	19M	4.2B	RegNetX-600MF [61]	6.2M	0.6B
EfficientNet-B5 [43]	30M	9.9B	RegNetX-800MF [61]	7.3M	0.8B
EfficientNet-B6 [43]	43M	19B	T2T-ViT-19 [62]	39M	8.4B
EfficientNet-B7 [43]	66M	37B	T2T-ViT-24 [62]	64M	13B

**Fig. 3.**
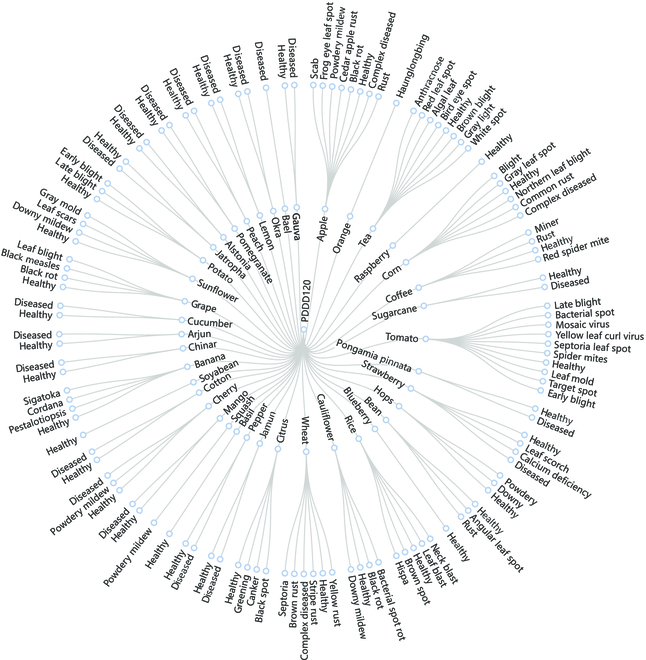
Taxonomy of the PDDD dataset.

After more than 2 years of collection, our large-scale PDDD includes over 400,000 images of plant diseases and 120 categories, as shown in Fig. 3 and Table 2. Our proposed large-scale plant disease dataset contains a wide range and diversity of plant diseases, allowing the plant disease pre-trained model to have adequate knowledge of plant diseases. As a result, computer vision technology will be applied more frequently in agriculture, and plant disease diagnosis will advance even further.

### Plant disease pre-trained model

#### Selection of pre-trained models

According to some review papers [38–41] in plant disease recognition and some trends in computer vision, we collect more than 60 categories of commonly used pre-train model structures. We first compile a list of common model structures to find the best pre-trained models for plant disease diagnosis. As shown in Table 4, we gather a variety of structural models such as AlexNet [8], VGGNet [9] GoogleNet [10], ResNet [21], DenseNet [42], EffecientNet [43], and others that can be used directly for plant disease classification or as backbone networks for other more complex models [44,45] to implement different tasks such as plant disease detection and segmentation. Furthermore, to facilitate the use of plant disease pre-trained models in plant disease detection and segmentation tasks, we collect the backbone network structures of the YOLO (You Only Look Once) series, MaskRCNN, FCN (Fully Convolutional Network), and DeepLab as part of the list of the pre-trained models. Since the Transformer architecture has achieved astounding results in computer vision over the last 2 years, we add VIT and Swin-Transformer to the list of pre-trained plant diseases to enable the Transformer architecture to advance the field of plant diseases. We hope that the subsequent work on plant disease diagnosis can appreciate the progressive nature of the transformer-based architecture. Furthermore, because the number of parameters in the pre-trained model influences time and accuracy, we list the plant disease pre-trained models in the table by the number of parameters to aid in future use. As shown in Table 3, users can achieve generalization from mobile portable devices to laboratory precision diagnosis for different scenarios by selecting plant disease pre-trained models with different parameters in Table 3.

**Table 4.** Superparameter and environment settings during training.

Particular	Description
Operating system	CentOS 7.9
Deep learning framework	Pytorch 1.8
GPU accelerated environment	CUDA 11.6
GPU	NVIDIA A100
CPU	Intel(R) Xeon(R) Silver 4314 CPU @ 2.40GHz
Mean value	(0.416, 0.468, 0.355)
Standard deviation	(0.210, 0.206, 0.213)
Batch size	32
Optimizer	Adam
Learning rate	0.01
Epoch	100

#### Model training

**Fig. 4.**
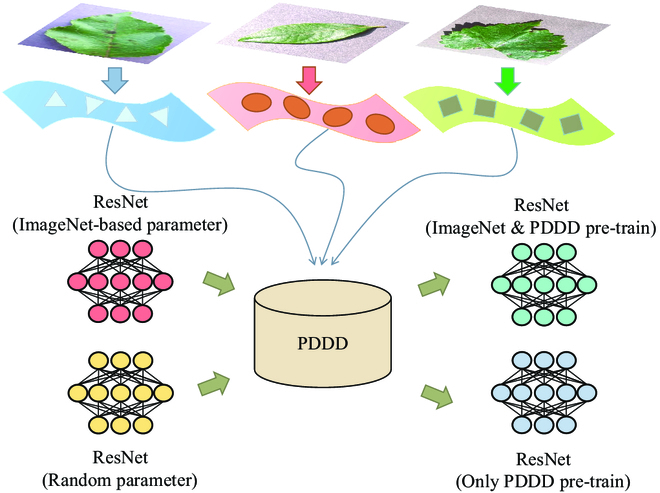
The role of pre-trained models in plant disease diagnosis.

Based on the collected pre-train model, we adopt some common tricks to train the models on our dataset, ensuring these models can fully absorb the knowledge of plant diseases. First, we set the training, validation, and test set ratios in the collected large-scale plant disease dataset to 8:1:1 and randomly assigned the images. All the images are deflated and center-cropped to a pixel value of 224 × 224 to fit the model input. Then, the models are trained on the dataset and utilized in various network structures to produce a pre-trained model of plant diseases with sufficient domain knowledge. Because there are some differences between the plant disease dataset and the ImageNet dataset, as shown in Table 4, the mean and standard deviation of the entire plant disease dataset must be recalculated. Additionally, we implement 2 training techniques for the pre-trained model for plant diseases to more precisely evaluate the effect of domain knowledge on the model. As shown in Fig. 4, one method is to train the model directly on the large-scale plant disease dataset using random parameters to ensure that the model only obtains professional knowledge from the dataset. The other is to adopt the weights from ImageNet to train transfer learning on a large-scale plant disease dataset so that the model not only gains knowledge of plant diseases from the dataset but also gains powerful generalization ability from the ImageNet dataset. Finally, we can obtain the series of plant disease pre-trained models, which can assist in plant disease diagnosis. During model training, we used the same super parameters to ensure consistency in training, as shown in Table 4.

**Fig. 5.**
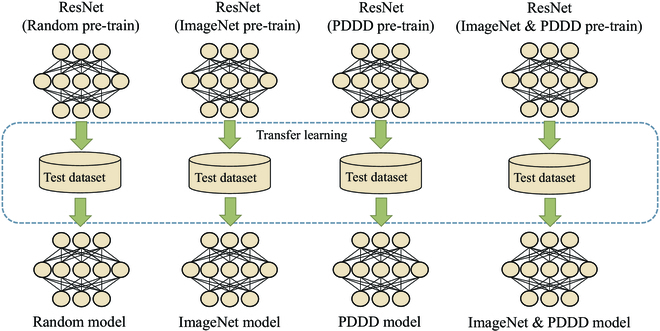
Four training methods with ResNet pre-trained models as an example.

#### How to use pre-model

We open-source all of our plant disease pre-trained models to improve the development of plant disease diagnosis and integrate computer vision methods with agriculture. Users can access these model weights via our links. Additionally, we maintain the structure as closely as possible to that in frequently used model papers to increase the usability of the pre-trained models for plant diseases. With only minor changes to the original code, users can use the pre-trained plant disease models for their datasets and tasks, leading to quicker and more precise diagnoses. In addition, users who do not have a dataset can directly use our plant disease pre-trained model for common plant disease recognition. Our model has excellent recognition accuracy for the 120 diseases used for training and can quickly recognize plant disease images provided by users. Users with their own datasets can use our pre-trained model for plant diseases through transfer learning. With a large amount of plant disease expertise in our model, users can diagnose plant diseases from their dataset and get better results than the original model. In addition, with the multiple types of models available on our website, users can choose the model that suits their environment to meet the different needs of plant disease diagnosis, from rapid detection in the field to accurate detection in the laboratory.

## Results

To demonstrate the level of advancement of our proposed plant disease pre-trained model, we conduct multiple experiments in the plant disease diagnosis tasks of plant disease identification, plant disease detection, and plant disease segmentation.

### Selection of pre-trained models

As shown in Fig. 5, we compare the performance of 4 pre-trained models on the test dataset, including a randomly generated parameter model, a pre-trained model with only the ImageNet dataset, a pre-trained model with only the large-scale plant disease dataset, and a pre-trained model with both the ImageNet and large-scale plant disease datasets pre-trained. The randomly generated parameter model is trained directly on the test dataset using the random parameter ResNet101 [21] model. The pre-trained model with only the ImageNet dataset is transferred to learn on the test dataset using the officially provided pre-trained weights. The pre-trained model using only a large-scale plant disease dataset is created by training the ResNet101 [21] model with random parameters directly on the plant disease dataset, then training it on the test dataset using transfer learning. The officially provided ImageNet pre-trained weights are combined with the extensive plant disease dataset to create hybrid pre-trained weights, which were used to create the pre-trained model. The hybrid pre-trained weights are utilized for training on the test dataset via transfer learning. As shown in Table 4, all experiments for the plant disease pre-trained model are performed on the same platform.

### Plant disease classification

Plant disease classification is a pivotal and prevalent task in plant disease diagnosis, wherein diseases are identified based on their phenotypes. With the advent of deep learning, plant disease identification by this technique is rapidly replacing traditional manual identification due to its cost-effectiveness and efficiency. Prevailing deep learning-based plant disease classification methods employ image multiclassification techniques and have achieved remarkable accuracy in existing plant disease classification tasks. These methods utilize neural networks to identify plant diseases in images by selecting the most similar categories among multiple labels.

Moreover, we incorporated a plant disease pre-training model based on the PlantVillage dataset and a hybrid pre-training model based on both the PlantVillage and ImageNet datasets into the plant disease classification task to confirm that the plant disease pre-training model learns prior knowledge from the dataset and to validate the effectiveness of the PDDD-based pre-training model. The training methodology of the pre-training model based on the PlantVillage dataset is similar to that of the PDDD-based pre-training model, except that the pre-training model based on the PlantVillage dataset is implemented by pre-training the PlantVillage dataset using random parameters. On the other hand, the hybrid pre-training model based on PlantVillage and ImageNet datasets is achieved by transfer learning of the official weight model on the PlantVillage dataset.

#### Evaluation metrics and validation dataset

For plant disease recognition, we adopt classification recognition accuracy as the evaluation criterion, and the accuracy is described as:

$$\mathrm{Accuracy}=\frac{T_{\mathrm{correct}}}{T_{\mathrm{sum}}}\times 100\%,$$

where *T_correct_* is the number of samples correctly classified for each diseased leaf type, and *T_sum_* is the total number of samples for that type of diseased leaf.

**Fig. 6.**
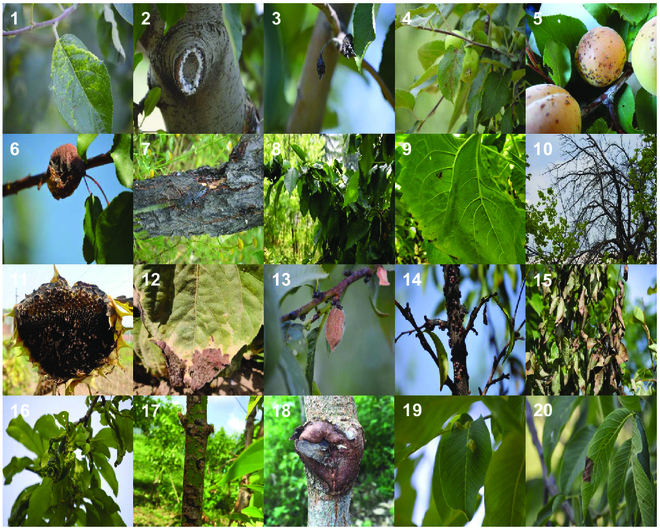
Example of images from the test dataset, representing every plant disease pair used. (1) Apple *Aphis* spp. (2) Apple *Eriosoma lanigerum*. (3) Apple *Monilinia laxa*. (4) Apple *Venturia inaequalis*. (5) Apricot *Coryneum beijerinckii*. (6) Apricot *Monilinia laxa*. (7) Cancer symptom. (8) Cherry *Aphis* spp. (9) Downy mildew. (10) Drying symptom. (11) Gray mold. (12) Leaf scars. (13) Peach *Monilinia laxa*. (14) Peach *Parthenolecanium corni*. (15) Pear *Erwinia amylovora*. (16) Plum *Aphis* spp. (17) RoughBark. (18) StripeCanker. (19) Walnut *Eriophyes erineus*. (20) Walnut *Gnomonia leptostyla*.

Our test dataset uses the plant disease dataset on Kaggle (https://www.kaggle.com/datasets/duggudurgesh/plantdataset), which has 5,106 images and 20 classes, as shown in Fig. 6. We choose this dataset for testing because the majority of its classes involve variations in plant phenotypic diseases rather than plant leaf diseases, demonstrating the portability and robustness of our pre-trained model as well as how our pre-trained model gains experience with plant diseases over time. We set the ratio of the training, validation, and test sets to 3:1:1 and centrally cropped to 224 × 224 to better fit our model for training.

#### Model selection

ResNet [21] has proven to be one of the most effective methods for image classification tasks, as it can capture rich and intricate information from images. This is achieved by increasing the depth of the neural network, which enables ResNet [21] to obtain more comprehensive image feature information. However, overly deep neural networks can result in the vanishing or exploding gradient problem, making it difficult to achieve optimal accuracy. To address this, ResNet [21] incorporates shortcut connections by using internal residual blocks, which alleviate the gradient problem associated with deep neural networks. Consequently, by increasing the depth of the network, ResNet can achieve greater accuracy than previous methods such as AlexNet [8] and VggNet [9]. Given the widespread use of ResNet [21] in plant disease classification, we chose ResNet-101 [21] as it strikes an excellent balance between model size and accuracy for plant disease classification comparison.

#### Comparison of pre-trained models

**Fig. 7.**
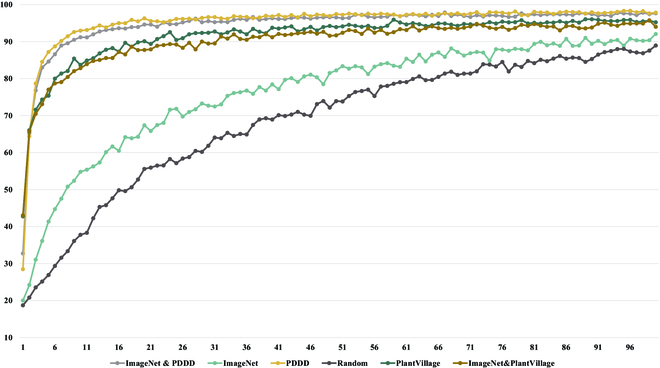
Comparison of the training accuracy of 6 pre-trained models on the test dataset.

**Fig. 8.**
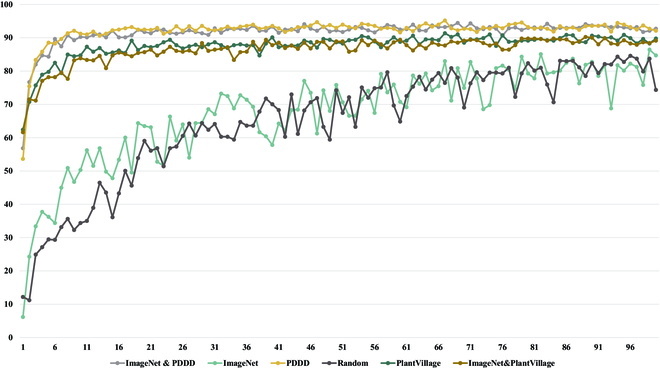
Comparison of the validation accuracy of 6 pre-trained models on the test dataset.

**Fig. 9.**
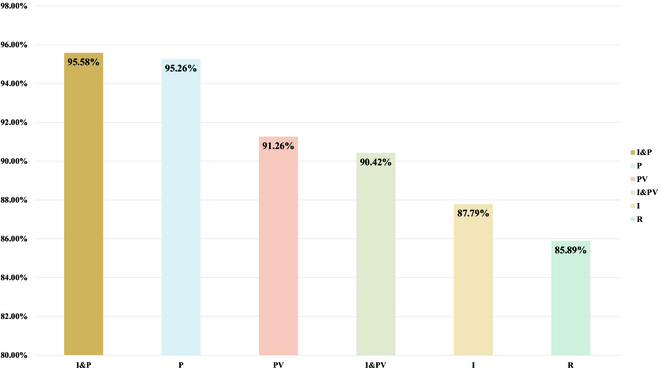
Comparison of prediction performance of 6 pre-trained models on test dataset (I&P: ImageNet&PDDD, P: PDDD, PV: PlantVillage, I&PV: ImageNet&PlantVillage, I: ImageNet, R: Random).

It is clear from the results shown in Figs. 7 to 9 that the proposed PDDD-based and PlantVillage-based plant disease pre-training models outperform the generic ImageNet-based pre-training models in terms of both training efficiency and accuracy. This suggests that the plant disease domain is specialized and has specific knowledge that is different from the generic domain. Thus, pre-training using plant disease datasets can provide more prior knowledge that is beneficial for plant disease diagnosis.

The hybrid pre-training model based on PlantVillage and ImageNet outperforms the pre-training model based on ImageNet alone in the plant disease classification task, indicating that using a dedicated plant disease pre-training model is more effective than using a generic pre-training model. However, the PlantVillage-based pre-training model was inferior to the hybrid plant disease pre-training model based on PDDD and ImageNet, indicating that pre-training using only the plant disease dataset may not fully learn all the necessary knowledge due to its smaller size compared to ImageNet. Therefore, there is a need to build a large-scale plant disease dataset.

Moreover, the PDDD-based pre-training model outperforms the PlantVillage-based pre-training model, indicating that a larger-scale plant disease dataset can provide more prior knowledge of plant diseases. Finally, the hybrid pre-training model obtained from ImageNet knowledge via migration learning performed the best, demonstrating that the higher applicability of pre-training models using mixed ImageNet and PDDD knowledge in classifying plant disease images.

### Plant disease detection

Plant disease detection is a technique for boxing diseased leaves in an image and predicting the disease category. Deep learning-based plant disease detection is primarily accomplished through object detection, where a neural network frames the diseased leaves in an image and then uses a classifier to predict the type of plant disease on the boxed leaves. Since the locations of plant disease images are mainly concentrated in the field with complex environments, the accuracy of plant disease images with complex backgrounds taken by using plant disease recognition processing exclusively could be better. However, plant disease detection is more suitable in complex environments by boxing out diseased leaves within an image and making category predictions. Additionally, plant disease detection offers the benefits of high real-time and high visibility, making it simpler to miniaturize and transport plant disease diagnosis. For these reasons, plant disease detection is one of the main future development directions of plant disease diagnosis.

#### Evaluation metrics and validation dataset

For plant disease detection, we use the mean average precision (mAP) of the average precision AP of each category as the evaluation index:

$$\mathrm{mAP}=\frac{1}{c}\sum_{c\in \mathrm{classes}}{\mathrm{AP}}_{c},$$

where *classes* is the set of categories supported by the model, *C* is the number of categories, and *AP_c_* is the AP of category c. *AP* is defined as follows:

$$\mathrm{AP}=\int_{0}^{1}‍P\left(R\right)dR,P=\frac{\mathrm{TP}}{\mathrm{TP}+\mathrm{FP}},R=\frac{\mathrm{TP}}{\mathrm{TP}+\mathrm{FN}},$$

where *TP* represents the number of positive samples detected, *FP* represents the number of negative samples detected incorrectly, and *FN* represents the number of positive samples not detected.

**Fig. 10.**
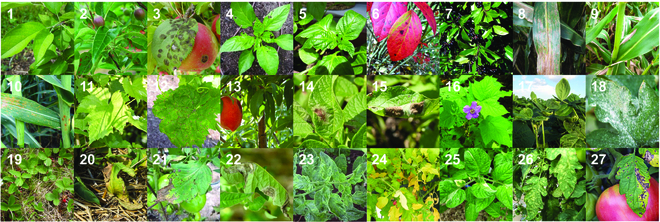
Example of images from the PlantDoc [46], representing every plant disease pair used. (1) Apple leaf. (2) Apple rust leaf. (3) Apple scab leaf. (4) Bell pepper leaf spot. (5) Bell pepper leaf. (6) Blueberry leaf. (7) Cherry leaf. (8) Corn gray leaf spot. (9) Corn leaf blight. (10) Corn rust leaf. (11) Grape leaf. (12) Grape leaf black rot. (13) Peach leaf. (14) Potato leaf early blight. (15) Potato leaf late blight. (16) Raspberry leaf. (17) Soyabean leaf. (18) Squash powdery mildew leaf. (19) Strawberry leaf. (20) Tomato early blight leaf. (21) Tomato leaf bacterial spot. (22) Tomato leaf late blight. (23) Tomato leaf mosaic. (24) Tomato leaf yellow virus. (25) Tomato leaf. (26) Tomato mold leaf. (27) Tomato septoria leaf spot.

We train and test the object detection model on a public dataset, PlantDoc [46], a multi-crop, multi-disease image classification, and object detection dataset covering 13 crops and 17 diseases, which should have 27 categories with a total of 2,598 images. We randomly select some images as a display, as shown in Fig. 10.

#### Model selection

**Fig. 11.**
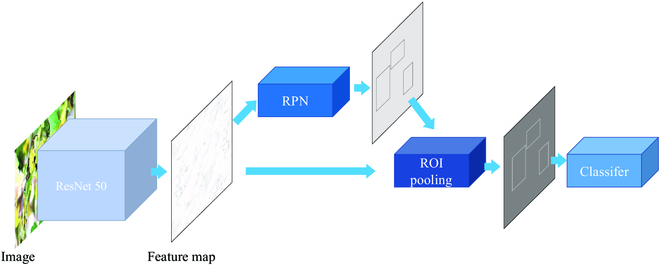
The main structure of Faster RCNN.

Faster R-CNN is one of the most accurate object detection models and can quickly generate high-quality region proposals. Compared to earlier 2-stage object detection models like R-CNN and Fast R-CNN, Faster R-CNN performs noticeably faster and more accurately. It also has higher accuracy than earlier single-stage object detection models like SSD (Single Shot MultiBox Detector) and YOLO. Faster R-CNN works in 2 stages: the first stage involves creating anchor rectangles from the input images and twice classifying the background and the target; the second stage involves regressing the rectangles and classifying the targets even more. Faster R-CNN achieves higher detection accuracy because it uses progressive refinement, a working method similar to humans’ detection of plant diseases. As shown in Fig. 11, Faster R-CNN has 5 major components: the feature extractor, region proposal network, region of interest pooling, classifier, and bounding box predictor. The complex structure complicates the faster R-CNN training process. However, an excellent pre-trained model can make the model’s training easy and thus make the model converge faster and better. Our work can provide better network initialization weights for the feature extractor of Faster R-CNN to reduce the network training difficulty. We choose the ResNet50 network as the feature extractor for Faster R-CNN so that the Faster R-CNN model can run on more computing-power-limited devices.

#### Comparison of pre-trained models

To verify the performance of our pre-trained models, we established 3 sets of experiments:

1. Faster R-CNN with random initialization (Random).

2. Faster R-CNN with the backbone’s initialization weights pre-trained from the large-scale plant disease dataset (Only PDDD).

3. Faster R-CNN with pre-trained backbone initialization weights from ImageNet and the large-scale plant disease dataset (PDDD & ImageNet).

We train and test them on PlantDoc using the same training hyperparameters, and we split PlantDoc [46] into a training set and a test set in a ratio of 1:10. Additionally, we used the model weights of the models trained in Only PDDD and PDDD & ImageNet as the initialization weights of the Faster RCNN. We increased the number of model training iterations to investigate the effects of model weights pre-trained by a large-scale plant disease dataset and model weights pre-trained by ImageNet and a large-scale plant disease dataset.

**Fig. 12.**
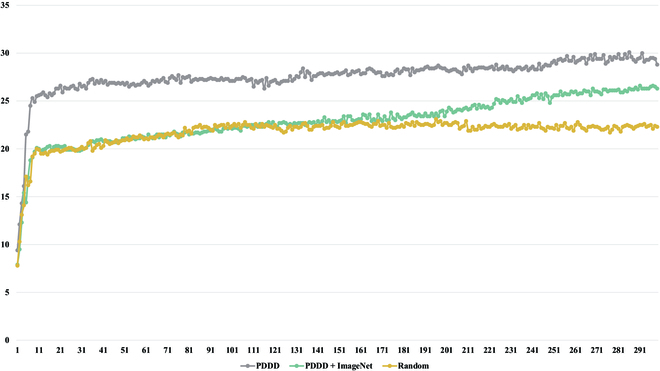
Comparison of the training accuracy of different pre-trained models on the PlantDoc [46] dataset.

**Fig. 13.**
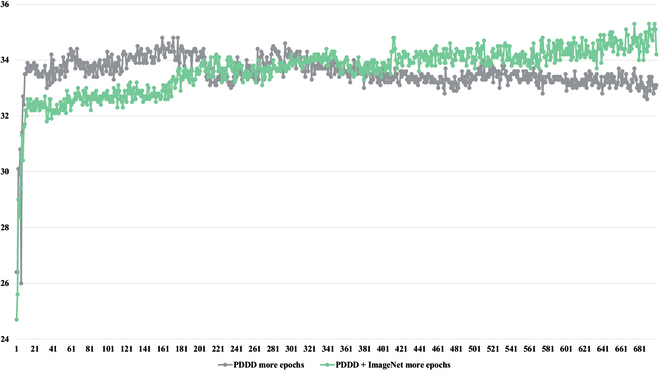
Comparison between 2 pre-trained models with more training iterations.

**Fig. 14.**
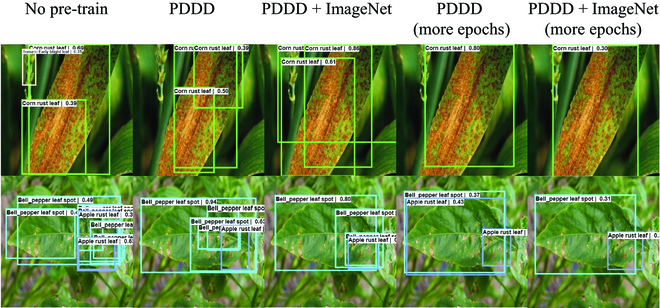
Comparison of the results using different pre-trained models.

As shown in Fig. 12, the detection accuracy and convergence speed of Only PDDD and PDDD & ImageNet are significantly better than those of Random, demonstrating that the pre-trained weights we provided can give the model practical knowledge about plant diseases, and this knowledge directs the model in the appropriate direction for optimization. Furthermore, as shown in Table 5 and Fig. 13, although Only PDDD converges faster than PDDD & ImageNet, PDDD & ImageNet can achieve a higher detection accuracy, and the detection accuracy of PDDD & ImageNet always increases, demonstrating a more robust capacity for generalization for the pre-trained model using the combined knowledge of ImageNet and PDDD. As seen in the following Fig. 14, the object detection model, which uses the pre-trained model we offered, can reduce the prevalence of incorrect identification, incorrect localization, and incorrect localization of plant diseases.

**Table 5.** Comparison of test accuracy of 5 pre-trained on PlantDoc [46] dataset.

Method	Random	PDDD	PDDD and ImageNet	PDDD (more)	PDDD and ImageNet (more)
mAP @.5	23.10%	30.10%	26.60%	34.80%	35.30%

**Fig. 15.**
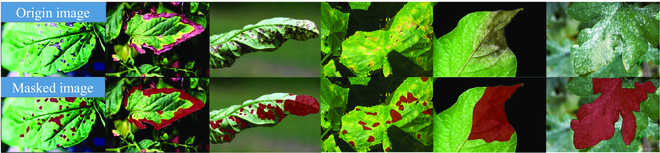
Example of images from the Kaggle, representing every plant disease pair used.

### Plant disease segmentation

Plant disease segmentation uses the pixel-level classification of disease locations in images to diagnose plant diseases. Deep learning-based plant disease segmentation is mainly implemented using semantic segmentation techniques. Since plant disease segmentation is a direct way to obtain the size of the leaf disease scale, this enables an easier understanding of the disease incidence than plant disease identification and detection. In addition, because the diagnostic accuracy of plant disease segmentation for leaves infected with multiple diseases at the same time is unmatched by other methods, plant disease segmentation is more suitable for more accurate diagnosis of plant diseases than identification and detection. In conclusion, plant disease segmentation is one of the crucial methods to achieve precision agriculture in the future.

#### Evaluation metrics and validation dataset

$$\mathrm{MIoU}=\frac{1}{k+1}\sum_{i=0}^{k}\frac{\mathrm{TP}}{\mathrm{FN}+\mathrm{FP}+\mathrm{TP}}$$

where *TP* represents the number of samples labeled positive and classified as positive and *FN* represents the number of positive samples classified as unfavorable. *FP* represents the number of samples labeled negative but classified as positive. Our test dataset is from the plant disease segmentation dataset on Kaggle (https://www.kaggle.com/datasets/fakhrealam9537/leaf-disease-segmentation-dataset), which contains 588 diseased leaf images and 588 masks of the corresponding images. The diseased leaf images are the leaves of several plants, and the masks of the corresponding images contain background and disease. We display some disease image examples, shown in Fig. 15.

#### Model selection

**Fig. 16.**
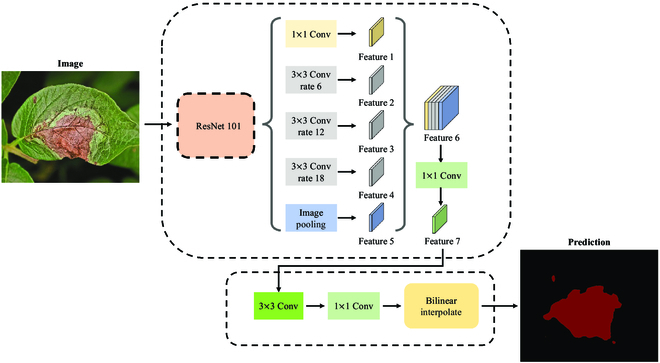
General structure of DeeplabV3.

DeeplabV3 is an excellent semantic segmentation model that revisits the role of dilated convolution in semantic segmentation. Among the Deeplab models, DeeplabV3 has drastically improved speed and accuracy over the previous DeeplabV2 and DeeplabV1. DeeplabV3 has a higher accuracy than other semantic segmentation models, such as PSPNet and SegModel. In addition, DeeplabV3 has a wide range of applications in plant diseases. Therefore, we choose the DeeplabV3 model as our experimental model. As shown in Fig. 16, DeeplabV3 makes several modifications based on DeeplabV2. To solve the multi-scale segmentation problem, DeeplabV3 designs a cascaded or parallel dilated convolution module that uses multiple atomic rates to capture multi-scale context and obtain more multi-scale contextual information. Secondly, DeeplabV3 enhances the ASPP (Atrous Spatial Pyramid Pooling) module, which detects convolutional features at multiple scales to enhance the global context of image-level feature encoding. In addition, the DeepabV3 model applies global averaging pooling on the last feature map, feeds the obtained features into a 1 × 1 convolution with 256 filters, and finally recovers the features to the desired spatial dimension by bilinear upsampling to further extract critical information. Finally, DeeplabV3 removes the DenseCRF post-processing operation of DeeplabV2, simplifying the model structure.

#### Comparison of pre-trained models

**Fig. 17.**
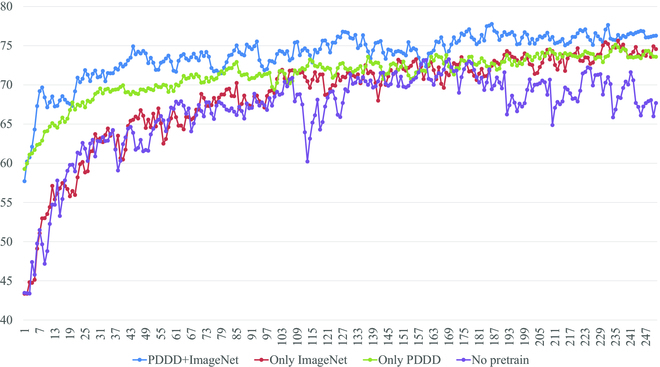
Comparison of the validation accuracy of different pre-trained models on the Kaggle dataset.

**Fig. 18.**
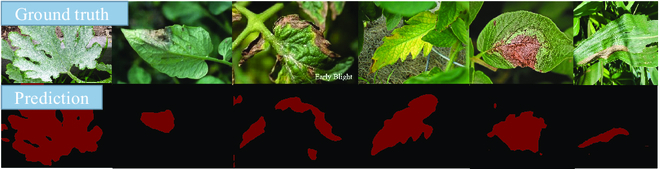
Semantic segmentation effect of plant diseases using DeeplabV3 with the PDDD pre-trained model.

We compare the 4 pre-trained models on the test dataset: the model initialized with random parameters, the pre-trained model with only the ImageNet dataset, the pre-trained model with only PDDD, and the pre-trained model with ImageNet and PDDD. These 4 models were trained and tested on the Kaggle public dataset, and our training and testing sets were divided into a 9:1 ratio for our models for comparison. The PDDD pre-trained models, as shown in Fig. 17, achieve excellent accuracy earlier than other pre-trained models during the training phase. This result indicates that the prior knowledge of plant diseases present in the PDDD pre-trained models may improve the ability of plant disease segmentation models to distinguish the disease’s location. Additionally, the plant disease pre-trained model that combined knowledge from PDDD and ImageNet consistently outperformed the other pre-trained models in terms of accuracy, demonstrating the potential of the pre-trained model to enhance the accuracy of the plant disease segmentation model.Figure 18 depicts the DeeplabV3 network’s resulting graph of our plant disease pre-trained model with mixed knowledge from PDDD and ImageNet for semantic segmentation of disease images. According to Fig. 18, the plant disease pre-trained model’s segmentation of disease regions in images matches the actual situation. According to the circumstances, the model, which uses the PDDD series pre-trained model, may help disease segmentation networks differentiate between different disease locations and assist in detecting plant disease severity.

## Discussion

Pre-trained models support the advancement of plant disease diagnosis methods and the gradual popularization of deep learning techniques in plant diseases. The proposed plant disease pre-trained model considerably reduces training time as well as improves model accuracy without changing the structure of existing diagnostic models. Additionally, our proposed plant disease pre-trained model provides a range of models for rapid diagnosis in portable devices to accurate diagnosis in the laboratory, offering an array of models for various plant disease diagnosis tasks. These will make an important contribution to the advancement of current precision agriculture.

Pre-trained models are frequently used in plant disease diagnosis methods based on deep learning, and some studies have recognized the full potential of these models. However, the related studies [47] did not pursue this phenomenon further, so the potential of pre-trained models in plant disease diagnosis remains unexplored. Our proposed pre-trained model yields promising results for plant disease diagnosis. Using a large-scale plant disease dataset, we provide sufficient plant disease domain knowledge to the pre-trained model. Meanwhile, we provide generic knowledge from ImageNet to the pre-trained model, and combining these 2 types of knowledge allows the plant disease pre-trained model to obtain a more robust plant disease feature representation. The excellent results also demonstrate that our point of view is authentic and trustworthy.

Plant disease diagnosis equipment varies according to use scenarios, and different plant disease diagnosis models have different requirements for different equipment. Choosing plant disease diagnostic models appropriate for the equipment is critical for plant disease diagnosis. This paper offers various plant disease pre-trained models for selection following the various disease diagnosis tasks and devices. Increasing the variety and size of plant disease datasets is a crucial step to improving the performance of plant disease pre-trained models because the performance of these models is closely related to the dataset’s quality. Finally, because plant disease pre-trained models are used for more than just disease diagnosis, optimizing plant disease pre-trained models to make them applicable in precision agriculture is a potential research direction that can provide technical assistance for subsequent plant growth detection, crop yield monitoring, agricultural automation detection, and so on.

This paper presents a series of pre-trained models for plant disease diagnosis. These models are pre-trained with large-scale plant disease datasets and can be used to help existing plant disease diagnosis pre-trained models gain more plant disease expertise, thereby improving the models’ performance on plant disease diagnosis tasks. After the extended experiments, we have demonstrated that using plant disease pre-trained models can achieve higher accuracy in a shorter training time. In addition, to better meet the needs of plant disease diagnostic devices, we propose a series of pre-trained models that can meet needs ranging from simple and fast detection in portable devices to accurate detection in laboratories. Overall, the pre-trained models of plant diseases can promote the development of plant disease diagnosis technology.

## Data Availability

All data and codes are available on the website https://pd.samlab.cn/ and Zenodo platform https://doi.org/10.5281/zenodo.7856293.
